# Reduced-port robotic radical gastrectomy for gastric cancer: a single-institute experience

**DOI:** 10.1186/s12893-022-01645-5

**Published:** 2022-05-19

**Authors:** Chih-Yuan Wang, Yu-Hsien Chen, Ting-Shuo Huang

**Affiliations:** 1 Division of General Surgery, Department of Surgery, Chang Gung Memorial Hospital, Keelung Branch, No. 222, Mai-Chin Road, 20401 Keelung, Taiwan; 2 Department of Chinese Medicine, College of Medicine, Chang Gung University, Kwei-Shan, 259 Taoyuan, Taiwan; 3 Community Medicine Research Center, Chang Gung Memorial Hospital, 20401 Keelung, Keelung Taiwan

**Keywords:** Gastrectomy, Gastric cancer, Lymph node dissection, Robotic surgery

## Abstract

**Background:**

Reduced-port laparoscopic gastrectomy can potentially reduce postoperative pain and improve recovery time. However, the inherent difficulty caused by the narrow manipulation angle makes this operation difficult, especially during lymph node dissection. The intrinsic advantage of the da Vinci^®^ robotic system might offset this difficulty, maintaining adequate surgical quality with risks of surgical complications equal to those by the conventional four-port robotic approach. The aim of this study was to compare the reduced-port robotic approach and the conventional four-port approach in terms of postoperative pain and short-term surgical outcomes.

**Methods:**

All patients who underwent radical gastrectomy with D2 lymph node dissection using the da Vinci Xi robotic system, including reduced-port or conventional four-port approach, were analyzed retrospectively. The primary outcome was postoperative pain assessed using the numerical rating scale (NRS). The secondary outcomes were the number of harvested lymph nodes, operation time, length of hospital stay, and postoperative 30-day complications.

**Results:**

Forty-eight patients were enrolled in the study, 10 cases in the reduced-port and 38 in the conventional four-port group. Postoperative NRS revealed no significant difference between the reduced-port and conventional four-port groups [postoperative day (POD) 1: 4.5 vs. 3, *p* = 0.047, POD 3: 4 vs. 3, *p* = 0.178]. After propensity score matching, there were no significant differences in the median number of harvested lymph nodes, operation time, and length of hospital stay between the groups. The postoperative 30-day complications were more frequent in the conventional four-port group, but there was no significant difference compared with the reduced-port group after propensity score matching.

**Conclusions:**

Reduced-port robotic gastrectomy with D2 lymph node dissection might be comparable to the conventional four-port robotic operation in terms of postoperative pain, surgical quality, and short-term outcomes. However, further studies are required to confirm our results and clarify the advantages of the robotic reduced-port approach.

## Background

Minimally invasive surgery is widely performed as one of the treatment modalities for gastric cancer [1–3]. Currently, radical gastrectomy with adequate lymph node dissection can be accomplished by either the laparoscopic or robotic approach [4, 5]. However, long-term survival benefits of robotic surgery are unclear. Moreover, laparoscopic or robotic gastrectomy may result in less wound pain and faster recovery while maintaining the same morbidity as that of traditional open gastrectomy [6–8].

With advances in medical equipment and surgical techniques, reduced-port or single-incision laparoscopic gastrectomy has been attempted to reduce the operative trauma and achieve quicker recovery [9–11]. However, the inherent difficulty of performing the reduced-port or single-incision operation makes these operations difficult to learn. Since the first robotic gastrectomy in 2003, its advantages, such as the 3D view, tremor filtering, and endowrist instruments, have provided surgeons with the opportunity to overcome the technical limitations of the conventional reduced-port or single-incision laparoscopic surgery [12, 13].

In this study, we analyzed the safety and benefits of robotic reduced-port and conventional four-port gastrectomy by using the intrinsic advantages of the robotic system to eliminate surgical difficulty.

## Methods

### Patient and study design

Data of all patients with gastric cancer who underwent robotic radical gastrectomy from October 2016 to December 2020 at our hospital were retrospectively analyzed. The surgical cases were classified into two groups: the reduced-port approach with an umbilical single-port access device and conventional four-port approach. Patients with metastatic lesions found during the operation, intraoperative conversion to open surgery, or conversion to palliative surgery were excluded. The selection for the reduced-port approach or conventional port approach is mainly patient-directed after fully explaining the risk and benefits of the two different kinds of techniques. All patients’ demographic data and perioperative and postoperative outcomes were collected for analysis. The primary outcome was the patient’s pain score assessed using the numerical rating scale (NRS, 0–10). The secondary outcomes were the number of retrieved lymph nodes, operation time, length of hospital stay, and postoperative 30-day complications. Operation time was defined as the time from the start of the first wound incision to final wound closure, including robotic docking and console time. The severity of postoperative 30-day complications was assessed using the Clavien–Dindo classification. All gastrectomy procedures in these two groups were performed with standard radical resection with D2 lymph node dissection, followed by Billroth II or Roux-en-Y anastomosis reconstructions. All operations in the robotic reduced-port and conventional four-port groups were performed by two surgeons, who also played the roles of console surgeons and assistants. This study was approved by the relevant institutional review board and all informed consent was provided to each patient.

### Robotic reduced-port and conventional four-port procedures

#### Operation method

**Fig. 1:**
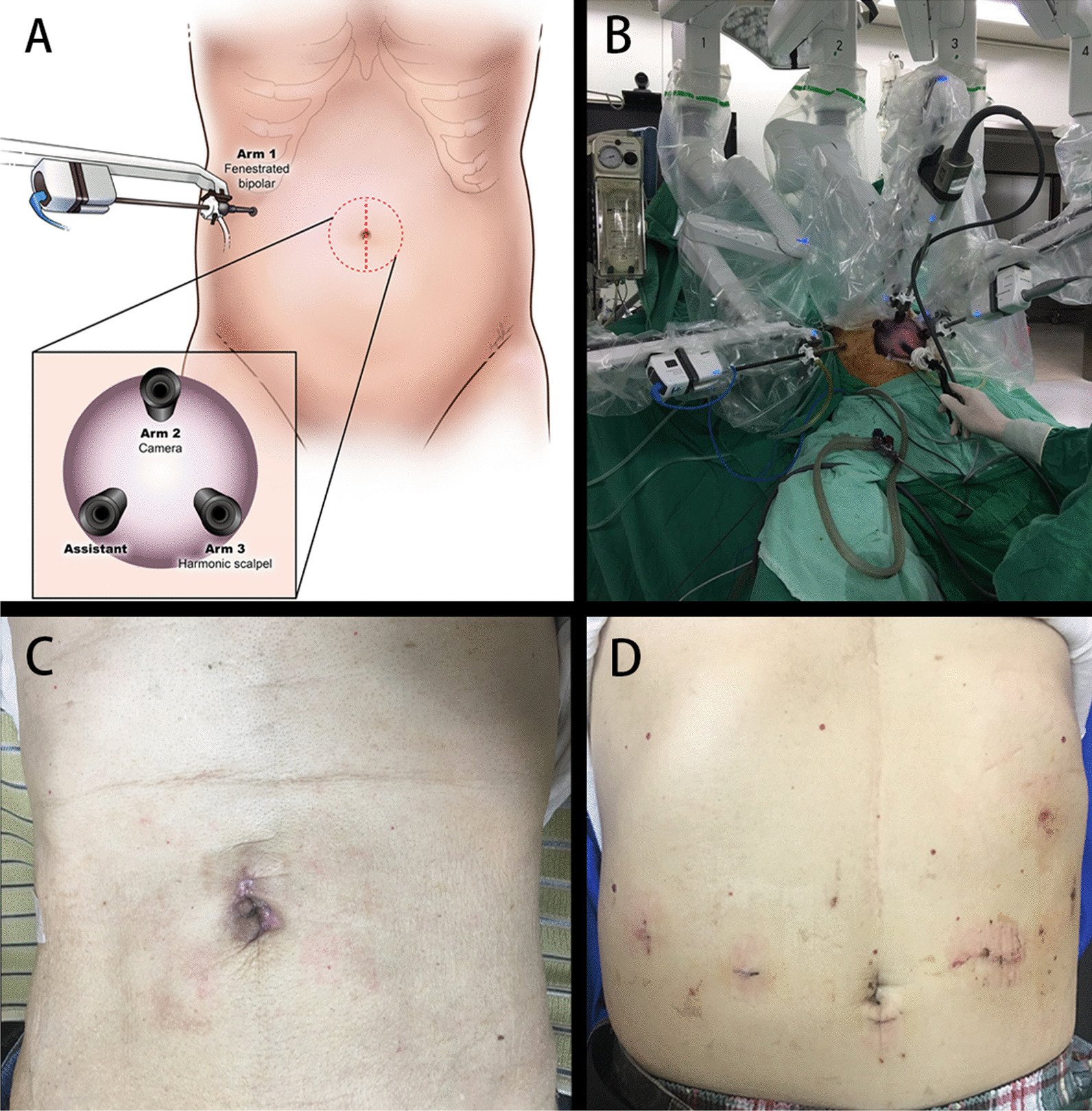
Robotic reduced-port approach. **A** One trans-umbilical vertical wound sized 3–4 cm for a single-port access system and one 8-mm trocar wound of the right abdomen. **B** The assistant is positioned between the patient’s legs. All hemoclip, Hem-o-lok, and endostapler instruments are utilized via the assistant’s laparoscopy instrument. **C** Final wound appearance. **D** Final wound appearance in the robotic conventional four-port approach

All robotic operations were performed using the da Vinci^®^ / da Vinci^®^ Xi™ Surgical System (Intuitive Surgical, Sunnyvale, CA, USA). The patient was placed in the reverse Trendelenburg position with bilateral leg spilt, and the assistant stood between the legs. The robotic Xi system was set up on the left side of the patient, leaving more space for the anesthesiologist. In the robotic reduced-port group, only two port wounds were created on the patient’s abdomen: a 3–4-cm vertical transumbilical incision and an 8-mm trocar wound located over the patient’s right lateral abdomen (Fig. 1A and C). After creating the transumbilical wound, the commercial single-port access system (Gelport or Gloveport) was inserted into the umbilical wound to maintain pneumoperitoneum and facilitate manipulation. The robotic fenestrated bipolar instrument was placed into the 8-mm trocar. The robotic harmonic scalpel, robotic camera, and laparoscopic assistant instrument were all obtained from the umbilical single-port system (Fig. 1B). On the other hand, in the conventional four-port group, a total of four port wounds were made. All trocar wounds were 8 mm in size, except the infraumbilical trocar wound that was 12 mm and used as the assistant port (Fig. 1D). Pneumoperitoneum was maintained using carbon dioxide insufflation with a target of 12 mmHg. Distal or total gastrectomy was performed in a standardized manner with D2 lymphadenectomy. In the reduced-port group, the gastric specimen was extracted from the umbilical single-port system before reconstruction. In the conventional four-port group, the infraumbilical incision was extended to 3–4 cm for extraction of the resected specimen. The reconstruction was performed after confirmation of negative proximal and distal margins of the resected specimen by intraoperative frozen examination. In the reconstruction period, the umbilical wound of the conventional four-port group had already been extended to 3–4 cm, which is the same size as that in the reduced-port group (Fig. 1C). The video of reduced-port total gastrectomy could be found on this weblink: https://youtu.be/lGAXFto-9cg.

#### Postoperative care

All patients who underwent gastrectomy received standard postoperative care in our hospital, which included early removal of the nasogastric tube on postoperative day 1 or 2 if there were no contraindications. Between the final skin closure and the endotracheal tube extubation, the anesthesiologist wound administered 1ml Fentanyl for post-operative pain control. After extubation, the patient would be transferred to the post-operative recovery room for about 1 h and then moved to the ward. Early feeding was initiated from sipping water to a scheduled post-gastrectomy diet depending on the patient’s clinical response. In addition, no routine intramuscular or intravenous analgesics were administered to patients if the pain score did not exceed NRS 3. After removing the nasogastric tube, oral analgesics were administered routinely for pain control once the patient resumed regular daily activities.

### Statistical analysis

All demographic and clinicopathological characteristics were summarized using descriptive analysis, and continuous data were compared using the two-tailed Student *t* t-test if normality holds. Otherwise, the Wilcoxon rank-sum test was performed. Categorical variables are presented as numbers and percentages and were compared using the Pearson χ^2^ test or Fisher’s exact test between the groups. Statistical significance was set at *p*-value of < 0.05. All analyses were performed using R version 3.6.3 (R Foundation for Statistical Computing, Vienna, Austria) with “MatchIt.”

## Results

A total of 48 patients were enrolled in the study. After propensity score matching, 10 and 20 patients were included in the reduced-port and conventional four-port groups, respectively. Patient demographics and clinical outcomes before and after matching are shown in Table 1. There were no significant differences in age, sex, and body mass index between the groups. The operation and reconstruction method used, clinical stage, and pathological stage were also not significantly different between the groups.

**Table 1:** Demographic data before and after propensity score matching of patients who underwent robotic reduced-port and conventional four-port gastrectomy

	Before propensity matching			After propensity matching		
	Reduced- port	Conventional four-port	*p* values	Reduced- port	Conventional four-port	*p* values
Patient, n	10	38		10	20	
Age, median [IQR]	70 [64,78]	74 [62,81]	0.732	70 [64,78]	70 [63,78]	0.947
Sex, female, n	2 (20)	18 (47)	0.16	2 (20)	5 (25)	1
BMI, median [IQR]	23 [22, 25]	24 [21, 25]	0.99	23 [22, 25]	23 [21, 26]	0.965
C stage, n, I	3 (30)	15 (40)	1	3 (30)	5 (25)	1
II	2 (20)	5 (13)		2 (20)	4 (20)	
III	5 (50)	18 (47)		5 (50)	11 (55)	
Total LN retrieved [IQR]	41 [29, 45]	31 [24, 40]	0.264	41 [29, 45]	31 [28,39]	0.28
Metastatic LN	2 [0, 14]	1 [0, 7]	0.568	2 [0, 14]	1 [0, 6]	0.682
p stage, n I	2 (20)	15 (39.5)	0.365	2 (20)	7 (35)	0.696
II	3 (30)	5 (13.2)		3 (30)	4 (20)	
III	5 (50)	18 (47.4)		5 (50)	9(45)	
Operation method, n			1			1
Distal gastrectomy	7 (70)	29 (76.3)		7 (70)	14 (70)	
Total gastrectomy	3 (30)	9 (23.7)		3 (30)	6 (30)	
Operative time, min, median [IQR]	450 [398, 473]	420 [360, 525]	0.684	450 [398, 473]	437 [365, 535]	0.912
Anastomosis method			1			0.693
BII	7 (70)	25 (66)		7 (70)	11 (55)	
Roux-en-Y	3 (30)	13 (34)		3 (30)	9 (45)	
Blood loss, ml			0.666			1
> 50 cc	8 (80)	32 (84.2)		8 (80)	15 (75)	
< 50 cc	2 (20)	6 (15.8)		2 (20)	5 (25)	
LOS, day, median [IQR]	16 [12, 22]	15 [11, 20]	0.859	16 [12, 22]	14 [11, 17]	0.659
Post-op complication			1			1
< Grade III^a^	10 (100)	35 (92.1)		10 (100)	19 (95)	
≥ Grade III	0 (0)	3 (7.9)		0 (0)	1 (5)	
NRS 1, median [IQR]	5 [4, 6]	3.5 [2, 5]	0.068	4.5 [4, 5.75]	3 [2, 4.25]	0.047
NRS 2, median [IQR]	4 [3, 5]	3 [2, 4]	0.2	4 [3, 5]	3 [2, 4]	0.2
NRS 3, median [IQR]	4 [2, 5]	3 [2, 4]	0.131	4 [2, 5]	3 [2, 4]	0.178

^a^Including no complication, grade I and II postoperative complication

The NRS scores on postoperative days 1, 2, and 3 between the two groups revealed no significant difference. In the reduce-port group, the NRS on postoperative day 1 was slightly higher than that in the conventional four-port group (4.5 vs. 3, *p* = 0.047). The NRS scores on postoperative days 2 and 3 between the two groups were similar (4 vs. 3, *p* = 0.178).

The total number of retrieved lymph nodes was higher in the reduced-port group than in the conventional four-port group, but the difference was not significant (*p* = 0.28). At least 28 lymph nodes were retrieved from both groups. The median operation times of both groups were similar (450 vs. 437 min, *p* = 0.912), and blood loss was also comparable. The median length of hospital stay in the two groups was 16 vs. 14 days (*p* = 0.659), and the longest hospital stay was 23 days.

After propensity score matching, grade 0, I, and II postoperative complications between the two groups were similar (10 vs. 19) (Table 1). Two cases in the reduced-port group had grade II complications: minimal esophagojejunostomy anastomotic leakage without any drainage intervention and mild aspiration pneumonia due to food choking. Prior to propensity matching, there were two grade IIIa and one grade IV complications in the conventional four-port group. The grade IIIa complications comprised intra-abdominal abscess requiring percutaneous drainage and acute kidney failure case requiring temporary hemodialysis without any permanent sequalae. One patient experienced grade IV complications due to sudden-onset ischemic stroke and required transfer to the intensive surgical care unit for further treatment. This patient recovered well thereafter and was discharged on postoperative day 23.

No mortality was noted within 90 days after surgery. There was no significant difference in complications in the 30 days after surgery between the two groups after propensity score matching. Table 2 lists in detail the complications of all cases prior to propensity score matching.

**Table 2:** Postoperative complications before propensity score matching

Clavien–Dindo classification	Reduced-port	Conventional four-port
I	1	9
II	2	9
IIIa	0	2
IIIb	0	0
IV	0	1
Details of complication		
Excessive abdomen pain	1	0
CVP infection	0	1
Pneumonia	1	4
Gastro-jejunal anastomotic leakage	1	0
Esophagojejunal anastomotic leakage	0	1
Dysuria	0	1
Nausea and vomiting	0	2
Delirium	0	2
Ischemic stroke	0	1^a^
Temporary hemodialysis	0	1^b^
Asthma attack	0	1
Intra-abdominal infection	0	2
Intra-abdominal abscess	0	1^b^
Delayed gastric emptying	0	1
Elevated liver enzymes	0	1
Postoperative ileus	0	2

^a^Grade IV, ^b^Grade III. *CVP* central venous catheter

## Discussion

In this study, we reported that robotic reduced-port gastrectomy is not inferior to conventional four-port gastrectomy in terms of short-term surgical outcomes and surgical quality. Meanwhile, we demonstrated an almost equal number of harvested lymph nodes between the two procedures. The reduced-port robotic gastrectomy used in our research might be applied to early gastric cancers and some selected advanced gastric cancers.

There were no significant differences in operation time between the robotic two-port group and conventional four-port group; however, compared with previous studies, the operation time in our study was longer [14, 15]. The reason for this is that, in our reduced-port approach, only two abdominal wounds were created, and a total of three robotic arms, including a camera, were utilized for tissue dissection and reconstruction. Moreover, we used this approach not only in the early stage but also in advanced stage gastric cancer cases, if suitable. However, most studies reported the use of three or more abdominal wounds or curved robotic instruments [14, 15]. In addition, regardless of the type of instrument used in the robotic system, extracorporeal instrument collision between the robot’s arms and the assistant’s hand occasionally exists in the umbilical single-port system area [14]. Furthermore, in our study, the reconstruction of BII or Roux-en-Y anastomosis was performed after the margin status was confirmed intraoperatively. Although this strategy will require a longer operation time, we believe that confirmation of the negative margin status before reconstruction cannot be compromised to attempt to reduce operation time.

The concept of reduced-port or single-incision surgery in the minimally invasive surgery field confers to the reduction of the number of port wounds or wound length to potentially improve postoperative pain, accelerate postoperative recovery, shorten length of hospital stay, and/or increase cosmetic satisfaction [9, 10, 16, 17]. Some studies have revealed that single-incision surgery may decrease postoperative pain, but this does not always result in quicker recovery or shorter hospital stay [10, 18–20]. In our study, the reduced-port group did not show any significant differences in postoperative pain and length of hospital stay compared with the conventional four-port group. The pain sensation of the abdominal wound decreases as the number of wounds or length of the wound decreases. In our study, compared with the reduced-port group, two extra 8-mm port wounds were created over the patient’s abdomen bilaterally in the conventional four-port group. Some studies reported that, compared with single-incision surgery, multi-port surgery resulted in creation of at least 2–4 extra port wounds of 12 mm and/or 5 mm in size on the abdomen [10, 20, 21]. Furthermore, different postoperative pain management strategies influence patients’ subjective experiences of pain differently. There are various factors that could influence incisional pain, including the number of ports, length of the incision, and individual characteristics [22]. However, the principle of reduced-port surgery was intended to reduce the number of ports without interfering with the quality of operation. In our study, the pain scores did not reveal any significant differences; therefore, the surgeon should try to achieve a balance between the number of wounds and the quality of operation.

The oncological outcome of gastric cancer surgery can be assessed by the number of retrieved lymph nodes [23–25]. Retrieval of a greater number of lymph nodes improves the staging accuracy of gastric cancer and survival due to increased clearance of nodal micrometastases [26, 27]. In our study, the reduced-port and conventional four-port groups yielded a median number of 41 and 31 lymph nodes, respectively, exceeding the recommended number of retrieved lymph nodes [23, 24]. Dissecting more lymph nodes may lead to increased bleeding; hence, dissecting lymph nodes without causing excessive blood loss or pancreas parenchyma injury is important when reaching the desirable number of retrieved lymph nodes [3].

Technically, robotic surgery can provide 3D images, endowrist articulation, and tremor filtering, which can increase the number of retrieved lymph nodes without causing excessive bleeding compared with laparoscopic surgery [8, 12, 18, 28]. Therefore, the intrinsic advantage of the robotic device makes robotic reduced-port surgery less technically demanding than the single-port or reduced-port laparoscopic surgeries, especially during lymph node dissection [18, 29, 30]. In other words, the difference in the degree of operative difficulty associated with tissue dissection between robotic reduced-port and conventional four-port surgeries might be less significant compared with the difference between reduced-port and conventional port laparoscopic gastrectomy.

In our study, regardless of the reduced-port or conventional port approach, only three robotic arms and two robotic instruments were used, including the fenestrated bipolar and harmonic scalpel for dissection. The only differences in these two approaches are the instrument approaching site and manipulation angle. In addition, the hemoclip, Hem-o-lok, or endostapler device were all applied via the assistant port using a laparoscopic instrument applier. Owing to the endowrist instrument and tremor filtering properties of the robotic equipment, intracorporeal dissection and reconstruction procedures were almost the same between the two groups, despite the different manipulation angles. Therefore, if the there was no significant difference in the NRS scores between the two groups, the subsequent recovery, length of hospital stay, and 30-day complications will theoretically have no significant differences between the groups. However, we did not measure the cosmetic satisfaction of the patient in either group, although theoretically, the cosmetic satisfaction will be slightly higher in the reduced-port group [9].

This study has several limitations. The number of patients in the reduced-port group was small. The main reason for this is the relatively higher cost of the robotic reduced-port procedure. Compared with the conventional port, additional US 1000 dollars were charged for the single-port access system advice. In addition, owing to the older age of the patients in our study, the possible cosmetic gain from the reduced-port procedure was somewhat neglected by the patients. We collected the data of both robotic total and distal gastrectomy cases because the purpose of the study was to compare robotic reduced-port to conventional robotic four-port surgery in terms of postoperative wound pain and short-term outcomes. The overall complications of the total gastrectomy might be relatively higher than that of the distal gastrectomy; however, the patient who received total gastrectomy operation between the two groups were similar in our study.

## Conclusions

Our study revealed that the reduced-port robotic distal or total gastrectomy with D2 lymphadenectomy might be comparable to the conventional robotic four-port approach for selected patients. There were no significant differences in terms of postoperative pain, surgical quality, or short-term postoperative outcomes. However, further comparative or large-scale randomized control studies are required to confirm our results and clarify the advantages of the robotic reduced-port approach.

## Data Availability

All data generated or analysed during this study are included in this article.

## Abbreviations

NRS: Numerical rating scale
POD: Postoperative day
